# Lesser Trochanter Migration following Intramedullary Fixation of an Intertrochanteric Femur Fracture

**DOI:** 10.1155/2016/9348032

**Published:** 2016-02-22

**Authors:** Carlo Montoli, Cecilia Pasquali, Elia Paiusco, Vincenzo Pellecchia, Ettore Vulcano

**Affiliations:** ^1^Department of Orthopedics, Ospedale di Luino, 21016 Luino, Italy; ^2^Department of Orthopedics-Limb Lengthening & Complex Reconstruction, Hospital for Special Surgery, New York, NY 10021, USA

## Abstract

Intertrochanteric femur fractures are commonly observed in the elderly and may be associated with a complete fracture of the lesser trochanter in over 50% of cases. The migration of the lesser trochanter secondary to the psoas muscle contracture is a rare event. This case report presents a rare case of sudden groin pain three-week status after intramedullary fixation of a intertrochanteric femur fracture.

## 1. Introduction

Intertrochanteric femur fractures are commonly observed in the elderly and may be associated with a complete fracture of the lesser trochanter in over 50% of cases [1]. The migration of the lesser trochanter secondary to the psoas muscle contracture is an exceptional event. However this has been reported to cause pseudoaneurysm of the femoral artery or femoral nerve palsy [1–5].

This paper presents a rare case of sudden groin pain three-week status after intramedullary fixation of a intertrochanteric femur fracture.

## 2. Case Report

An 80-year-old woman presented to the authors' hospital emergency department complaining of right hip pain after sustaining a fall in her own home. Prior to injury she could walk normally. She had a normal body mass index and did not suffer from any other comorbidities. Clinical and radiographic evaluation of the right hip demonstrated an AO type 31.A2 intertrochanteric fracture.

**Figure 1:**
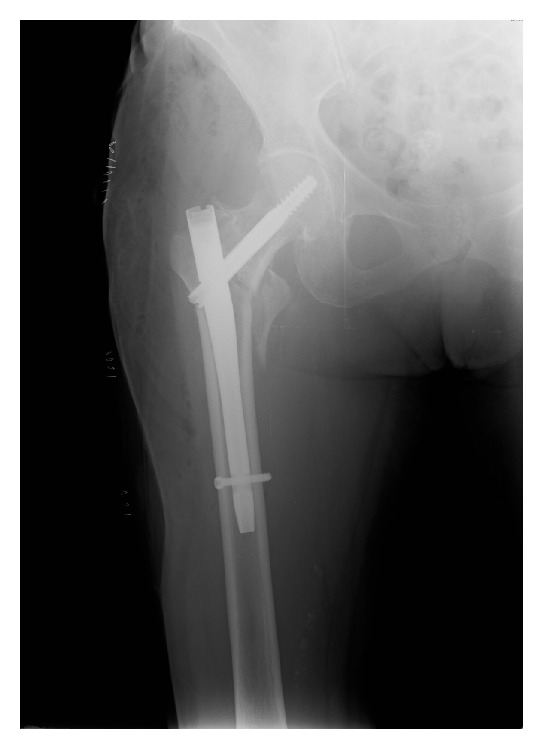
Immediate postoperative AP view radiograph of the right hip demonstrating good position of the anterograde intramedullary nail.

**Figure 2:**
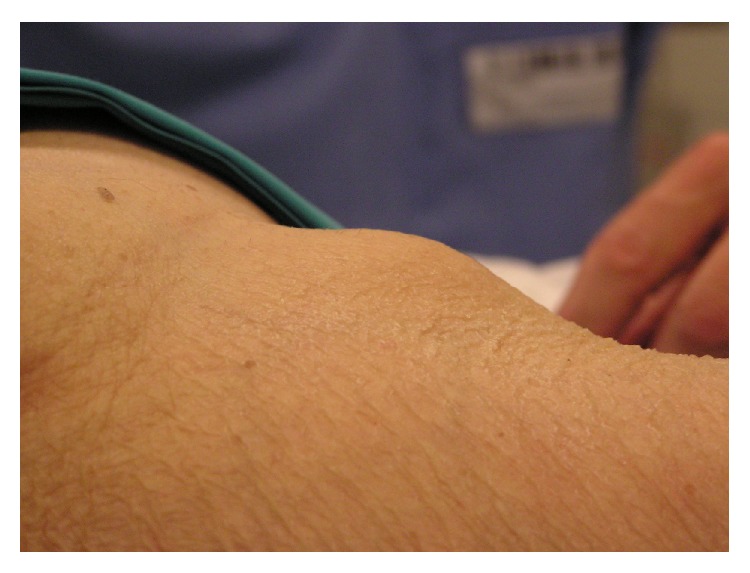
The photograph shows the visible right groin prominence that appeared at about 3 weeks postoperatively.

The femur fracture was treated with intramedullary fixation the following day (Figure 1). The patient tolerated the procedure well and the immediate postoperative period was completely uneventful. She was instructed not to bear weight for two weeks, but a range of motion exercises were started on postoperative day one. After an unremarkable hospital stay she was transferred to a rehabilitation facility on postoperative day four. Two weeks following surgical fixation of the femur fracture the patient started progressive weight-bearing and gait training. However, on postoperative day 20 the patient complained of a sudden onset pain at the right groin, over the femoral triangle of Scarpa. On physical examination the patient presented with a palpable and visible mass of about 3 × 3 cm, hard and tender to palpation (Figure 2). Arterial pulses, strength, and sensation of the right lower extremity were unaffected. The pain was poorly controlled by rest and pain medication.

**Figure 3:**
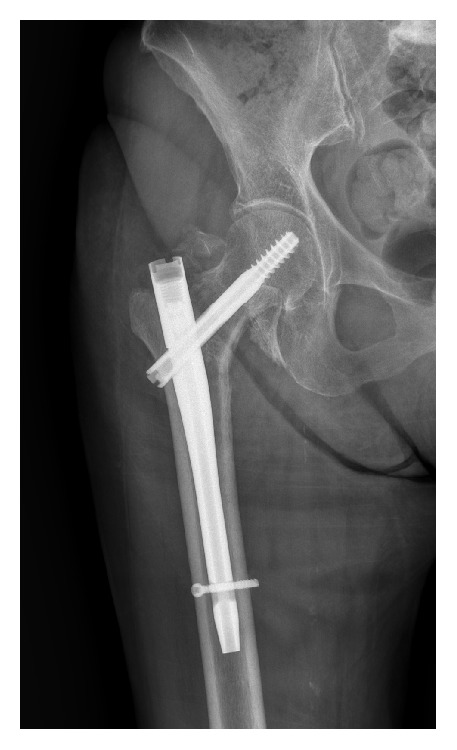
The AP view radiograph of the right hip demonstrates proximal migration of the lesser trochanter.

**Figure 4:**
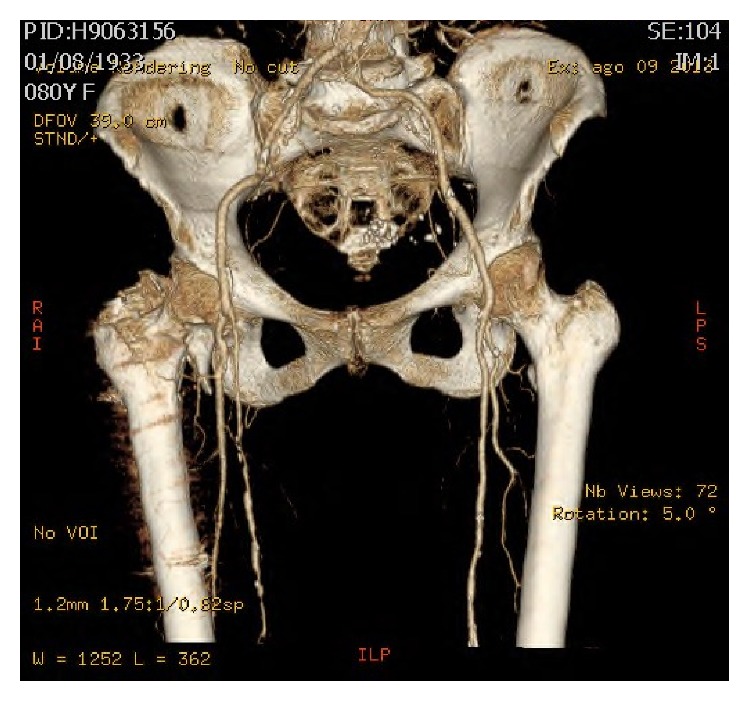
The angio-CT scan demonstrates proximal migration of the lesser trochanter in the femoral triangle of Scarpa without neurovascular involvement.

On postoperative day 40 anteroposterior and lateral X-rays were obtained demonstrating cephalad migration of the lesser trochanter (Figure 3). For such reason an angio-computed tomography (CT) was obtained to assess fragment location and femoral vascular involvement (Figure 4). The study demonstrated the migration of the lesser trochanter over the femoral triangle of Scarpa with no apparent neurovascular injury. On postoperative day 45 the patient underwent surgery for removal of the dislocated lesser trochanter. Intraoperatively the ascending branch of the lateral circumflex artery was isolated and retracted to access and remove the bony fragment. The apex of the fragment was medial to the deep femoral artery and distal to the lateral circumflex artery, both being completely intact. Further, there was no lesion/compression of the superficial femoral nerve, which has been described as the source of pain in a previous report [3]. It is likely that the complaint of pain was therefore caused by skin tension rather than nerve injury.

The patient tolerated the procedure well and the immediate postoperative period was uneventful. She was allowed immediate weight-bearing and was completely asymptomatic at four weeks postoperatively.

## 3. Discussion

Migration of the lesser trochanter after intramedullary fixation of a pertrochanteric fracture of the femur is extremely rare. These complications have been observed both preoperatively [2, 3] and after intramedullary fixation [1, 4, 5]. Four of these cases [1, 2, 4, 5] presented with either deep or superficial femoral artery injury, while one case [3] presented with femoral nerve palsy. The present study reports the first case of postoperative lesser trochanter cephalad migration manifesting as a subcutaneous painful groin mass and neurovascularly intact lower extremity.

Unfortunately there was poor communication between the rehabilitation facility and the orthopedic surgeons involved in the treatment of the patient. There was a substantial delay between the presentation of groin pain and the diagnosis of dislocation of the lesser trochanter to the femoral triangle. As pointed out in previous case reports [1, 2, 4, 5], this may have determined further life-threatening complications such as femoral artery bleeding. It is often the case that elderly patients treated for pertrochanteric hip fractures are transferred to rehabilitation facilities postoperatively. Most commonly this is determined by presence of comorbidities, impossibility of performing activities of daily living alone, and difficulty to reach physical therapist offices once discharged home. Rehabilitation facilities therefore play a crucial role in the overall care of posttraumatic patients. Nonetheless, health care operators of these facilities should be made aware of the possible postoperative complications, and orthopedists should have the responsibility of educating both patients and health care operators. Further, there should be more frequent and closer interaction between the surgeons and the rehabilitation facilities to prevent delays in diagnosis of any complication.

Patients with suspected migration of the lesser trochanter should be immediately investigated clinically to check for pulses and neurologic deficits and radiographically. Dislocation of the lesser trochanter should then be further studied with Doppler ultrasound and/or angio-CT scan to rule out femoral neurovascular injury. Finally, prompt surgical treatment should be considered based on both clinical and imaging findings to treat or prevent severe complications.

This study has the inherent weaknesses associated with a retrospective case report. However, it makes orthopedic surgeons aware of a possible rare complication that may present with one of the most common traumatic injuries in orthopedic surgery. This report not only alerts orthopedists to the possibility, but should also lead to accumulation of data over time. This case should affect preoperative discussions with patients or surgical planning.
